# Oral Health Care Services, Barriers and Enablers to Maintaining Good Oral Health in Motor Neurone Disease: A Scoping Review

**DOI:** 10.1111/cdoe.70061

**Published:** 2026-03-11

**Authors:** Mariam A. Khokhar, Lucy A. O'Malley, Anne‐Marie Glenny, Xiaohui Chen

**Affiliations:** ^1^ Division of Dentistry, School of Medical Sciences The University of Manchester Manchester UK

**Keywords:** motor neurone disease, oral health, oral hygiene, toothbrushing

## Abstract

**Objectives:**

The objective of this scoping review was to map existing literature on oral health and related care in individuals with Motor Neurone Disease (MND). Specifically, the review aimed to identify barriers and facilitators to maintaining oral hygiene, summarise available clinical guidelines and patient‐facing resources, and examine how oral health care is integrated within multidisciplinary management of MND.

**Methods:**

The review focused on oral health practices without restrictions on language, publication date or study type, excluding studies unrelated to MND or oral health. Data sources included MEDLINE, Embase, CINAHL, and grey literature such as clinical guidelines and patient resources. Screening and data extraction were performed independently by two reviewers to ensure rigor.

**Results:**

Of 847 studies screened, eleven primary studies met the inclusion criteria, comprising case reports, case series, self‐reports, cross‐sectional studies and letters. The grey literature search identified three clinical guidelines and eight patient information leaflets/resources. The included studies spanned diverse populations, including Amyotrophic Lateral Sclerosis (ALS) patients with varying disease subtypes and care needs, and explored oral hygiene difficulties, care barriers and unique insights from the case studies. Identified gaps highlighted the lack of integration of dental professionals into multidisciplinary care teams. Barriers such as physical limitations, caregiver dependency and limited‐service accessibility were prevalent. However, caregiver involvement, multidisciplinary collaboration and innovative solutions like antimicrobial photodynamic therapy and adaptive oral aids emerged as enablers. Poor oral health was strongly associated with increased pain, aspiration pneumonia and diminished well‐being, emphasising the need for targeted interventions.

**Conclusion:**

Embedding oral health management within multidisciplinary care frameworks for MND patients, enhancing caregiver training, improving access to dental services and adopting innovative strategies will improve patient outcomes and inform future research.

## 1 Introduction

Motor neurone disease (MND) is “a fatal, rapidly progressing disease that results in degeneration of the motor neurones, or nerves, in the brain and spinal cord” [1]. Over time, MND results in progressive muscle weakness and atrophy, leading to reduced limb mobility as well as impairments in speech, swallowing, and respiration [1]. The global prevalence of MND is estimated at 3.4 per 100 000 population [2] although prevalence varies geographically. Higher prevalence rates have been reported in Europe (up to 6.0 per 100 000), Australasia, and North America, whereas lower rates are observed in sub‐Saharan Africa and parts of Asia [2, 3]. These variations may reflect genetic, environmental, or diagnostic differences across populations. Global incidence rates average around 0.78 per 100 000 person‐years, with approximately 57 000 new cases diagnosed annually [2].

Approximately one‐third of individuals with MND die within the first year of diagnosis, and over half within 2 years [4]. Additionally, cognitive and behavioural changes occur in up to 50% of individuals, with some developing frontotemporal dementia.

Although MND does not directly affect oral health, physical impairments and limited access to care pose challenges to maintaining oral hygiene, adversely affecting health and quality of life [5]. For instance, loss of upper limb function, fatigue and wasting of muscles also makes brushing of teeth and oral hygiene management difficult [5]. MND also affects the gums and teeth by indirectly affecting a person's ability to maintain their oral hygiene effectively. This often leads to poor gum health, decaying of teeth and high rates of tooth extraction [6]. Similarly, bulbar weakness (affecting the muscles of the tongue, jaw, and throat) result in dysphagia, oromotor dysfunction, pooling of saliva and aspiration making it difficult to use mouthwash or toothpaste risking perioral infections [7].

MND care is typically met through a multidisciplinary team (MDT) that includes neurologists, specialist nurses, dieticians, physiotherapists, occupational therapists, speech and language therapists, respiratory physiologists and palliative care specialists [1, 8]. Current National Institute of Care Excellence (NICE) guidelines [1] emphasise MDT coordination with links and prompt access to additional services such as clinical psychology, social care, respiratory ventilation services, orthotics and gastroenterology. Although NICE guidelines highlight the need for oral health management in terms of nutrition, gastrostomy, and for those with saliva issues, they provide limited guidance and the dental team is not a part of the MDT or associated services. There are no details regarding what “support, advice and interventions” should be provided for oral health or by whom, leaving non‐oral health specialists to address oral health issues without relevant expertise.

Similarly, the Mouth Care Matters programme developed by Health Education England (now a part of NHS England) in 2015 promotes hospital‐based oral care [9] and the importance of hospital staff training and education on the efficacy and safety in delivering mouth care. However, the programme does not specifically address MND‐related challenges, either for home‐based or residential care. While there may be parallels with other conditions, individuals with MND face unpredictable symptom progression which complicates oral hygiene routes for both patients and their caregivers [10].

Therefore, this scoping review aimed to explore how oral health and related care are addressed in the context of Motor Neurone Disease. The specific objectives were to:

1. Identify barriers and facilitators influencing oral hygiene maintenance among individuals with MND;
2. Map existing clinical guidelines, information leaflets and patient resources related to oral health in MND.

## 2 Methods

The scoping review was conducted according to a published protocol which can be found here: https://doi.org/10.17605/OSF.IO/VDCEZ.

### 2.1 Inclusion Criteria

Studies focusing on oral health care services, oral hygiene practices or the oral health needs in individuals affected by MND or Amyotrophic Lateral Sclerosis (ALS), including those with related conditions such as bulbar palsy or muscular atrophy, were included. The population of interest extended to caregivers, dental professionals, speech therapists and other stakeholders involved in managing oral health for MND/ALS patients. Both primary and secondary data sources, including quantitative and qualitative studies, were eligible; studies unrelated to MND or ALS or those not addressing oral health or oral care services were excluded.

### 2.2 Search Strategy

The search for sources of evidence encompassed both peer‐reviewed articles and grey literature. Specific sources included MEDLINE (Ovid), Embase (Ovid), Cochrane Library, Epistemonikos and CINAHL; trial registries ClinicalTrials.gov and the WHO International Clinical Trials Registry Platform; and the study protocol database PROSPERO. Searches were undertaken October 2024 Grey literature was sourced from OpenGrey, National Technical Information Service (NTIS), charity websites, guideline development bodies, and other organisations with unpublished studies. A comprehensive search strategy was developed, including keywords related to oral health, tooth diseases, gingivitis, mouth diseases and oral hygiene, combined with terms for MND and ALS. There were no restrictions on the language and publication date, and no study filters were applied. Table 1 describes the search strategy used.

**TABLE 1:** Search Strategy (Medline OVID) (search run: 7.10.2024).

1.	oral health.mp. or exp. Oral Health/
2.	exp Tooth Diseases/
3.	(caries or “tooth decay”).mp.
4.	ginigivitis.mp.
5.	mouth diseases/or burning mouth syndrome/or candidiasis, oral/or lichen planus, oral/or mucositis/or oral ulcer/or exp. periodontitis/or tooth loss/or tooth migration/or tooth mobility/or stomatitis/
6.	exp Oral Hygiene/
7.	toothbrushing.mp. or Toothbrushing/
8.	mouthcare.mp.
9.	dental care/or dental care for aged/or dental care for chronically ill/or dental care for disabled/
10.	Dental Devices, Home Care/
11.	(dental and floss$).mp.
12.	(“motor neuron disease” or “motor neurone disease” or MND or “amyotrophic lateral sclerosis” or ALS or “bulbar palsy” or “muscular atrophy”).mp.
13.	exp Motor Neuron Disease/
14.	1 or 2 or 3 or 4 or 5 or 6 or 7 or 8 or 9 or 10 or 11
15.	12 or 13
16.	14 and 15

Two team members (AMG and MAK) screened an initial sample of studies and discussed findings to ensure consistency. Screening of titles and abstracts was then undertaken independently and in duplicate, with full‐texts being retrieved for any study identified by at least one reviewer. Full‐text screening was independently conducted by two reviewers (AMG and MAK), with any discrepancies resolved by a third reviewer (LM) through discussion.

A data charting form was developed to extract relevant data including study characteristics, details of oral health care services and oral hygiene practices, oral health needs, barriers and enablers. Table 2 describes characteristics and main findings of the primary studies included in this scoping review.

**TABLE 2:** Characteristics and main findings of primary studies included in the scoping review.

Author (Year)	Country	Study design/Type	Sample/Participants	Oral health care services	Oral hygiene practices	Oral health needs	Impacts of poor oral health	Enablers	Key barriers
Asher & Alfred 1993	USA	Case report	A 25‐year‐old male diagnosed with amyotrophic lateral sclerosis (ALS) at age 21. The patient was followed for 5 years at the University of Colorado School of Dentistry's Special Care Clinic	Extensive dental care over 5 years, including multiple restorations and extractions. Procedures were performed upright using local anaesthesia to minimise aspiration risk. Fluoride applications were largely ineffective due to nausea and gag reflex sensitivity	Minimal dental decay before ALS onset; oral hygiene declined with disease progression due to reduced muscle control. Despite preventive care, plaque accumulation, caries and occasional self‐inflicted oral injuries	Required extensive treatment for caries, gingival inflammation and fractured cusps. Oral health declined due to reduced muscle control and poor oral clearance, necessitating regular monitoring and ongoing dental care	Increased dental caries following ALS onset due to food retention and reduced oral muscle activity. Desire to maintain oral sensations contributed to plaque accumulation and self‐inflicted tongue/lip injuries	Primary caregiver support (by his mother) was essential for communication and access to dental care. Long‐term management was facilitated through the University of Colorado Special Care Clinic	Severe gag reflex, dysphagia and respiratory issues required cautious treatment planning to prevent aspiration. Many clinics declined care, highlighting limited professional awareness of ALS‐specific dental needs
Birgitta Bergendal and Anita McAllister 2017	Sweden	Case series	Fourteen ALS patients (9 women, 5 men; mean age 62.8 years) were included. Participants were grouped by disease onset: bulbar (*n* = 8) and spinal (*n* = 6). The study took place at the National Oral Disability Centre for Rare Disorders, Jönköping, Sweden	Patients received orofacial and oral care assessments every 3 months to support oral health during disease progression. Baseline examinations guided the use of oral aids and professional cleaning. Additional tools (electric toothbrushes, tongue scrapers, shields, retractors, swabs) were introduced as needed. Care was delivered by a multidisciplinary dental team comprising dentists, hygienists and assistants	Most patients used electric toothbrushes, and all required assistance with oral hygiene towards the later stages of the disease. Family members or personal assistants helped the patients with tooth brushing and cleaning routines. Tongue scrapers were used to reduce plaque buildup on the tongue and oral swabs were used to moisturise the mouth and apply fluoride solutions	Key oral health needs included maintaining hygiene to prevent decay and managing ALS‐related orofacial dysfunctions. Bulbar‐onset patients showed greater chewing, swallowing, drooling and speech difficulties, requiring specialised oral care support	Reduced orofacial function, particularly tongue immobility, led to plaque buildup, debris and tongue coating, increasing risks of halitosis and aspiration pneumonia. Bulbar‐onset patients developed severe dysfunctions earlier, greatly limiting their ability to maintain oral hygiene	Family and caregiver support was essential for maintaining oral hygiene, aided by regular dental visits and use of oral aids under multidisciplinary guidance. Sweden's dental insurance provided free care for ALS patients, eliminating financial barriers to ongoing oral health management	Progressive muscle weakness, especially in bulbar‐onset patients, reduced the ability to perform independent oral hygiene. Communication impairments further limited patients' ability to express oral health needs or discomfort
Eliziário Vitoriano de Araújo Neto Jr., Alinne Patierry Oliveira Pacífico Feitosa, Giovanni Iury Martins Pontes, Luiz Carlos Costa Madeira Alves. 2018	Brazil	Case Report	The study is a case report focusing on a 51‐year‐old Brazilian woman with amyotrophic lateral sclerosis (ALS). The patient was maintained in permanent home care hospitalisation and presented with a deep carious lesion on a permanent tooth (tooth 31).	The patient received minimally invasive home‐based dental treatment involving partial caries removal and antimicrobial photodynamic therapy (aPDT). Local anaesthesia and manual curettes were used, with methylene blue and low‐level laser applied to disinfect the cavity	The patient likely required substantial assistance with oral hygiene due to advanced ALS and home‐bound status. A minimally invasive treatment approach was prioritised given her inability to access routine dental care	The main oral health need was management of a painful deep carious lesion while avoiding complex procedures due to medical fragility. ALS‐related functional decline necessitated specialised dental care and assistance with oral hygiene	The deep carious lesion caused significant pain and reduced quality of life, with potential for infection or tooth loss if untreated. Advanced ALS limited access to routine dental care, increasing the risk of unmanaged oral disease	Antimicrobial photodynamic therapy (aPDT) enabled conservative, home‐based dental treatment, minimising the need for complex procedures. Access to professional dental home care was essential, preventing deterioration of oral health in the absence of clinic‐based treatment	ALS‐related immobility required a home‐care approach, restricting access to conventional dental services. Although cognition was intact, physical limitations created challenges in pain control, communication and oral hygiene management
Alessandro de Sire, 2021	Italy	Cross sectional study	The study included 37 ALS patients (mean age 61.2 years), comprising 10 with bulbar onset and 27 with spinal onset disease	All patients received multidisciplinary care involving neurologists, rehabilitation physicians and oral health specialists. Oral assessments included BOHSE, WTCI, OFDI and GI, while overall function was measured using ALSFRS‐R	Most patients exhibited poor oral hygiene and specific daily care routines were not described. The study highlighted the need for structured oral care and rehabilitation within ALS management	Key needs included managing sialorrhea, tongue immobility and maintaining oral hygiene. Bulbar‐onset patients experienced greater tongue movement limitations, making self‐care particularly difficult	Poor oral health was linked to lower functional scores, shorter survival and increased sialorrhea. Elevated WTCI scores indicated more tongue coating and bacterial buildup, heightening risks of aspiration pneumonia and infection	Integrating oral health professionals into ALS care was emphasised to improve overall health and survival. Non‐invasive ventilation (NIV) was noted to benefit oral health by reducing saliva production and sialorrhea‐related complications	Progressive loss of mobility and oral motor function hindered routine hygiene even with assistance. A lack of structured oral health protocols in multidisciplinary ALS management was identified as a major service gap
Moulding & Koroluk 1991	USA	Case Report	A 67‐year‐old woman with progressive bulbar palsy (a subtype of ALS) presented with a two‐year history of dysarthria, dysphagia and excessive drooling	The patient was referred for management of chronic drooling unresponsive to anticholinergic therapy. A custom heat‐cured acrylic lip plumper prosthesis was fabricated to improve lip seal and fluid retention, effectively reducing drooling	The patient demonstrated good self‐maintained oral hygiene with no notable periodontal disease. Preserved upper limb function enabled independent oral care despite partial tooth loss	The main oral health need was controlling drooling and fluid leakage while preventing lip trauma during eating. The lip plumper prosthesis improved lip competence and oral seal, resolving both issues	Chronic drooling caused social embarrassment and self‐image concerns, along with maceration at the mouth corners. Without intervention, ongoing skin irritation and psychological distress could further reduce quality of life	The lip plumper prosthesis successfully controlled drooling and lip biting, improving comfort and social confidence. It provided a non‐invasive, removable option that allowed the patient to maintain independence and oral hygiene	Progressive muscle weakness from bulbar palsy caused lip incompetence and dysphagia, worsening drooling and fluid loss. Pharmacological management with anticholinergics was ineffective due to adverse side effects
Nakayama 2018	Japan	Case report	The study involved 50 hospitalised ALS patients (31 men, 19 women; mean age 70.7 years) receiving tracheostomy positive‐pressure ventilation (TPPV) at Sayama Neurology Hospital, Saitama, Japan, between May and November 2014	Oral care was performed by nurses, including tooth brushing, tongue and mucosal cleaning, and saliva aspiration twice daily. No salivation‐reducing treatments were used. Assessments recorded mouth opening, dentition, periodontal status and tongue abnormalities	Nurses maintained patients' oral hygiene, though reduced mouth opening from ALS progression posed challenges. All participants were hospitalised and unable to perform self‐care due to advanced disease	The main oral health need was managing limited mouth opening (mean MMO 13.7 mm vs. 45–55 mm normal), which hindered oral care. Dental calculus and tongue anomalies further compromised oral hygiene	Limited mouth opening and poor oral health complicated routine care, heightening risks of infection and aspiration pneumonia. Tongue abnormalities were common, with 60% showing atrophy or hypertrophy and 52% exhibiting coating	Regular nurse‐led care maintained oral hygiene despite disease‐related limitations. Early dental intervention was recommended to manage declining mouth opening, while higher salivation rates may have helped protect against caries and gingival disease	Restricted mouth opening was the main barrier to effective oral care, limiting cleaning access and increasing disease risk. Tracheostomy and tube feeding further complicated hygiene by causing saliva pooling and reliance on aspiration
Rover & Morgano 1988	USA	Case study	A 64‐year‐old man with progressive bulbar palsy (ALS subtype), on anticoagulant therapy for thrombophlebitis and with comorbid depression, was non‐ambulatory	The patient developed a chronic self‐inflicted lower lip ulcer from repetitive chewing. Severe jaw reflexes and anticoagulant therapy limited treatment options, so a custom polyvinyl oral shield with elastic retention was fabricated to prevent trauma and allow easy cleaning	Daily oral hygiene was not detailed, as care focused on managing lip trauma. Nursing staff removed the shield at night and reapplied it each morning while monitoring for recurrent lip‐chewing	The main oral health need was to prevent self‐inflicted lower lip trauma from habitual chewing linked to bulbar palsy. A non‐invasive, biocompatible device was required to protect tissues without interfering with anticoagulant therapy	Chronic lip‐chewing led to ulceration and infection requiring IV erythromycin. The custom oral shield effectively prevented further trauma, with no recurrence of lesions during the last 2 months of life	The low‐cost, flexible oral shield offered an effective, non‐invasive solution for managing lip‐chewing. Its simple, replicable design allowed quick fabrication and replacement, ensuring continuous protection from self‐inflicted trauma	Severe jaw reflexes prevented conventional mouthguard fabrication, while anticoagulant therapy restricted invasive treatment options. Surgical interventions were avoided due to high complication risk
Tay & Borromeo 2014	Australia		The study involved 33 MND patients (both sexes) attending a multidisciplinary clinic in Melbourne, Victoria. Participants had advanced disease with varying presentations, including bulbar and limb onset types	Dental assessments were integrated into multidisciplinary MND care, evaluating dentition, caries, periodontal status and oral hygiene via Plaque and Gingival Indices. Ten of 27 dentate patients required extractions or restorations and three partial dentures needed adjustment	Self‐reported questionnaires captured dental attendance and hygiene routines. Eight participants reported regular dental visits, while others attended irregularly. Oral hygiene was generally maintained through daily brushing assisted by carers or nursing staff	Many participants required restorations for caries, retained roots, or fractured cusps. Dysphagia and bulbar symptoms complicated oral care, but no clear link between MND type and poor oral health was found, likely due to effective caregiver and multidisciplinary support	Common oral health issues included retained roots, caries and fractured cusps, causing pain, chewing difficulty and infection risk. Halitosis was observed in some patients, linked to poor hygiene and mouth breathing from respiratory impairment	Caregivers and nursing staff were essential in supporting oral hygiene for patients with limited mobility. The multidisciplinary clinic model ensured integrated dental and medical care, preventing oral health decline in advanced MND cases	Limited mobility and bulbar symptoms restricted access to dental care, leading to poorer oral health among irregular attenders. Fear of aspiration during treatment further discouraged some patients from seeking dental care
Van slems Maurits et al. 2024	Netherlands	Letter	The article addressed oral health challenges in ALS patients, particularly those with upper limb dysfunction limiting oral hygiene. It also referenced post‐mortem findings identifying aspiration pneumonia as a common cause of death in ALS	The article highlighted that oral hygiene is often neglected in ALS care by both professional and informal caregivers, increasing infection risk. It emphasised caregiver education and integrating oral health into multidisciplinary ALS management	Progressive hand and arm weakness in ALS increases reliance on caregivers for oral hygiene. An exploratory study cited revealed that oral care is often a low priority for both patients and caregivers, reflecting limited awareness and practice	The key oral health need is reducing oral microbial load to prevent aspiration pneumonia, a leading cause of death in ALS. The article stresses the importance of regular oral hygiene and calls for further research into ALS‐specific oral care strategies	Poor oral hygiene in ALS elevates the risk of aspiration pneumonia due to bacterial buildup and aspiration of pathogens. Although ALS does not directly affect the oral cavity, progressive disability hinders hygiene and contributes to systemic complications	Caregiver assistance becomes vital as ALS progression limits self‐care ability. The article advocates interdisciplinary collaboration among dentists, nurses and neurologists, supported by caregiver education to prioritise oral hygiene in ALS management	Progressive motor decline, especially in the hands and arms, limits patients' ability to maintain oral hygiene. Additionally, poor awareness and limited education among healthcare providers and caregivers hinder effective oral health management in ALS
Vaudroz et al. 2022	Switzerland	Case Report	This is a historical case report that analyzes the long‐term impact of amyotrophic lateral sclerosis (ALS) on the orofacial features of Stephen Hawking, based on publicly available photographs. It provides a rare opportunity to study the evolution of ALS over more than 50 years, focusing on the development of orofacial changes and malocclusions	The study does not report specific oral health care services administered to Stephen Hawking. Instead, it focuses on the photographic documentation of his facial and dental changes over several decades. The analysis reveals tooth loss, malocclusion and dental restorations but lacks detailed information on any dental interventions he may have received during his life. The authors speculate on potential dental treatments that might have been provided, including restorations and prosthetic work to address missing teeth, though the details of these treatments are not confirmed	The report does not discuss Hawking's oral hygiene practices, but it does observe the progressive deterioration of his dentition over time, which might indicate challenges in maintaining oral hygiene due to his ALS condition. The inability to perform regular oral hygiene could have contributed to the development of gingivitis, dental caries and tooth wear	he study highlights several oral health needs related to ALS, such as managing dysphagia (difficulty swallowing), tooth loss, malocclusions and muscle atrophy affecting the orofacial sphere. Over the years, Hawking experienced dental crowding, mandibular anterior proclination and a decrease in vertical facial dimension due to tooth loss, with mandibular anterior rotation leading to changes in his bite	The progressive deterioration of Hawking's teeth and loss of posterior dentition had significant effects on his facial profile, leading to a concave profile, mandibular protrusion and the increased prominence of the mandibular incisors. The authors suggest that the loss of oral function and deterioration of oral health could have impacted his overall quality of life, though this is speculative given the lack of medical details. The presence of dental caries, periodontal disease and attrition over time suggests that poor oral health may have been exacerbated by the challenges of ALS, including reduced muscle control and difficulties with oral hygiene	The study does not directly discuss enablers, but the multidisciplinary care Hawking likely received throughout his life may have included some level of dental support, particularly in the form of restorations and prostheses.	The key barriers identified include the progressive muscle weakness caused by ALS, which likely made it difficult for Hawking to maintain proper oral hygiene. This led to a gradual decline in his oral health over the years. Additionally, his respiratory needs and tracheostomy may have contributed to the challenges of receiving dental care or maintaining oral hygiene, particularly in the later stages of his illness
Makizodila 2022	Netherlands	Cross sectional survey study	The study included 259 patients with various forms of Motor Neuron Disease (MND), including Amyotrophic Lateral Sclerosis (ALS), Primary Lateral Sclerosis (PLS) and Progressive Spinal Muscular Atrophy (PSMA)	A digital survey was used to assess the oral health care needs of MND patients, their use of dental services and the level of support they received from dental professionals. 40.5% of patients expressed a desire to receive support from a dental professional for their oral care, while 71.8% reported never receiving information about the importance of oral health from their MND treatment team. Only a small percentage of patients visited a centre for special dental care (6.9%), while the majority (74.9%) visited a regular dental practice after their MND diagnosis	19.7% of patients were dissatisfied with their daily oral care, especially those who received assistance from caregivers. The study highlighted that many patients with reduced arm‐hand function struggled with maintaining their oral hygiene, leading to dependence on caregivers for oral care tasks such as brushing	The primary oral health need identified was support for daily oral hygiene, especially for patients with reduced motor function. Patients needed assistance with tasks such as brushing, flossing and maintaining denture hygiene, as they were often unable to perform these tasks independently due to physical limitations. The study also emphasised the need for better integration of oral care into the overall multidisciplinary management of MND	Poor oral hygiene in MND patients can lead to serious complications such as oral infections, gingival disease and aspiration pneumonia, which could further compromise their health. The study found that patients who did not ask for support with oral hygiene had a significantly worse oral health‐related quality of life (OHRQoL). Patients who were dissatisfied with their oral care had more difficulty using their arms and hands, which contributed to ineffective oral hygiene	Caregivers played a crucial role in assisting patients with oral hygiene. However, many caregivers expressed a desire for training from dental professionals to improve their ability to care for their patients' oral health. The study recommended increased collaboration between dental professionals and MND treatment teams to ensure that oral health is not overlooked in the management of MND. Educating patients and caregivers about oral hygiene could improve OHRQoL	The main barrier to effective oral care was the lack of support and guidance from dental professionals. Many patients were not adequately informed about the importance of oral hygiene in the context of MND, leading to neglect of oral care. Physical limitations due to disease progression also hindered patients' ability to perform their own oral hygiene. Additionally, some patients experienced difficulty in asking for help with their oral care, resulting in poorer outcomes

*Note:* This table follows the Community Dentistry and Oral Epidemiology journal format, combining study characteristics and main findings in a single comprehensive table.

The data extraction process was piloted on 2 papers and the findings discussed. Following this, data extraction was conducted independently and in duplicate (AMG, MAK, LOM); discrepancies were addressed through discussion (AMG, MAK, LOM, XC).

## 3 Results

**FIGURE 1:**
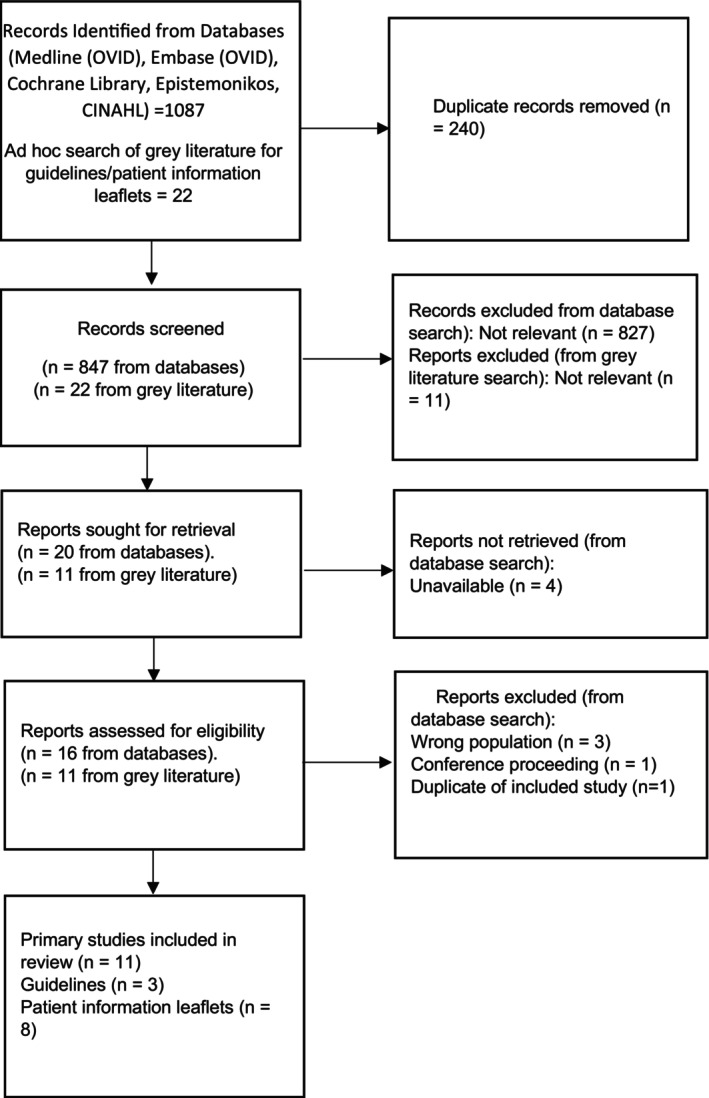
PRISMA 2020 flow diagram illustrating the study selection process. Adapted from Page et al. [21].

Of the 847 primary studies screened from electronic databases, eleven met the inclusion criteria, comprising case reports [7, 11, 12, 13, 14], case series [15], a self‐report [16], cross‐sectional studies [17, 18], a letter [19] and a case study [20]. Studies were conducted across various countries, with the majority originating from the United States, Sweden, Brazil, and Italy, highlighting the global interest in the intersection of oral health and neuromuscular disorders. Ongoing studies identified through searches of ClinicalTrials.gov and WHO international Clinical Trials Registry and PROSPERO have not all been listed in this scoping review. However, it was noted that the main area of research was the management of sialorrhea. The *ad hoc* grey literature search identified three clinical guidelines and eight patient information leaflets/resources, making the total number of included records twenty‐two. Figure 1 below provides the PRISMA flowchart for this scoping review in accordance with the PRISMA 2020 statement [21].

The included material was divided into: 1 primary research, 2 current guidelines and information leaflets specifically designed for patients, carers or health professionals to enhance knowledge and support care management.

### 3.1 Primary Research

The primary research identified eleven studies. The main themes that were identified in the studies were care approaches, oral hygiene practices, oral health needs, barriers and enablers.

#### 3.1.1 Care Approaches

Across studies, oral care for individuals with MND/ALS was characterised by a multidisciplinary and adaptive model, often involving collaboration between neurologists, rehabilitation physicians and dental teams [16, 17]. The integration of oral health into broader care frameworks was most successful when dental input was routine and proactive. Treatment plans were tailored to disease progression, emphasising minimally invasive and home‐based approaches where hospital attendance was not feasible [11]. Positioning strategies, such as maintaining the patient upright during procedures, and the use of local rather than general anaesthesia were critical adaptations to minimise aspiration risk and respiratory distress [7]. In countries like Sweden, systemic enablers such as state‐funded dental care [15] improved access and continuity, contrasting with settings where out‐of‐pocket costs restricted treatment.

#### 3.1.2 Oral Hygiene Practices

Oral hygiene practices evolved dynamically with disease progression. In early‐stage MND, patients often maintained independence using electric toothbrushes and adaptive aids, while later stages necessitated carer‐dependent hygiene, with family members or nursing staff performing twice‐daily cleaning and secretion management [13, 15]. Nurse‐led oral care in hospitalised or tracheostomy‐dependent patients was reported to maintain basic hygiene but often lacked professional dental oversight [13]. The use of adjunctive tools, tongue scrapers, oral swabs and fluoride rinses helped compensate for reduced oral clearance [15]. However, many studies observed plaque accumulation, halitosis, and tongue coating, particularly in patients with bulbar onset or restricted mouth opening [12, 22]. Consistent with these findings, the absence of structured oral‐care protocols in most multidisciplinary clinics led to variability in hygiene outcomes [18]. The findings collectively suggest that caregiver engagement, regular training, and standardised oral hygiene routines are critical for maintaining oral health as MND progresses.

#### 3.1.3 Oral Health Needs

The reviewed studies identified functional, preventive, and rehabilitative needs as central to maintaining oral health in MND. Functional needs included managing drooling, dysphagia, tongue immobility and lip incompetence, all of which affect mastication, speech and comfort [7, 15]. Preventive needs focused on reducing microbial load, calculus and caries, which are risk factors for aspiration pneumonia, a leading cause of mortality in MND [19]. Rehabilitative interventions aimed to preserve oral function and quality of life, using customised devices such as lip plumpers, oral shields, or modified dentures to restore lip seal and prevent self‐inflicted trauma [12, 20]. Studies from Japan and Brazil emphasised the value of home‐based dental interventions and photodynamic therapy to manage oral infections safely outside conventional settings [11].

#### 3.1.4 Barriers

The literature highlights numerous barriers at patient, procedural and systemic levels. Physical limitations, such as severe jaw reflex [20], jaw rigidity and reduced mouth opening [13] and severe gag reflex [13] undermined both self‐care and clinical interventions. Communication difficulties, especially among those with bulbar‐onset MND, limited patients' ability to report oral discomfort or request assistance [11, 15]. Fear of aspiration also deterred patients from seeking care [12]. Systemic challenges included insufficient training among carers, fragmented care pathways and inadequate access to special‐care dental services, particularly for home‐bound or ventilated individuals [7, 18]. Several studies also noted the psychological burden of dependence and embarrassment due to drooling, which compounded avoidance of dental care [14]. Collectively, these barriers underscore the urgent need for integrated oral health frameworks within MND multidisciplinary care.

#### 3.1.5 Enablers

Family members and carers provided essential daily oral care, often guided by informal instruction or professional oversight [7, 15, 18]. Multidisciplinary integration [16, 17] ensured timely interventions, with dental hygienists collaborating alongside neurologists and speech therapists [13]. The use of adapted tools such as electric toothbrushes, tongue scrapers, and saliva aspirators facilitated safer, more effective hygiene despite declining motor control [16, 20]. In some settings, policy enablers, including publicly funded or domiciliary dental services, removed financial and mobility barriers [15].

### 3.2 Current Guidelines and Information Leaflets

The scoping review identified three key guidelines offering formal recommendations for oral health management in MND: *NICE Guidelines* [1], *MND Saliva Control Guidance* [21], and *European Academy of Neurology (EAN) guidelines* [22]. Eight information leaflets provided guidance for patients, carers, and healthcare professionals on oral health in MND. Key resources included the MNDA information guides (e.g., *Motor Neurone Disease: Information for Dental Teams* [5], *Managing Dysphagia in Motor Neurone Disease* [23], *Managing Saliva Problems* [24] and *Caring for person with MND: a guide for care workers* [25]) as well as additional materials from MND Australia [26], Metro North Hospital and Health Service (*Mouth Care and MND*) [27], ALS Canada [28] and ALS Belgium [29]. Each resource addresses critical aspects of saliva management, oral hygiene maintenance and dysphagia but varies in its approach, emphasis, and level of detail.

#### 3.2.1 Saliva Management

Saliva‐related symptoms, including sialorrhea, xerostomia, and thick secretions, are common in MND and significantly impact patient comfort, respiratory health, and quality of life. Clinical guidelines and patient information leaflets consistently emphasise comprehensive assessment, considering saliva volume, viscosity, swallowing function, diet, posture and respiratory status [1, 5, 22, 23, 26].

Pharmacological interventions form the cornerstone of clinical management. First‐line treatments typically include antimuscarinic agents such as glycopyrronium bromide and hyoscine hydrobromide [1, 22, 23]. For refractory cases, botulinum toxin injections are recommended [22, 23], and the EAN guidelines additionally propose radiotherapy as a last‐resort option [23], a recommendation echoed in some international leaflets [30]. Other anticholinergic agents, including scopolamine and amitriptyline, are noted as alternatives in specific contexts [22, 23].

Non‐pharmacological strategies are widely advocated, particularly in caregiver‐facing materials. These include maintaining hydration, using mucolytics (e.g., carbocisteine) and nebulised saline, and implementing dietary modifications, such as reducing dairy and caffeine intake [23, 27, 30]. The use of suction devices and postural adjustments was frequently advised to help manage excessive drooling and thick saliva [22, 26, 27]. Some resources also reference natural remedies for mucus management [30] and Mechanical Insufflation‐Exsufflation (MI:E) devices, which assist secretion clearance in individuals with ineffective cough. These require respiratory specialist support and caregiver training [26].

#### 3.2.2 Oral Hygiene in Motor Neurone Disease (MND)

Maintaining oral hygiene in MND was a consistent priority across both clinical guidelines and caregiver‐oriented materials due to its critical role in preventing infections such as aspiration pneumonia and oral thrush. Progressive motor decline, bulbar dysfunction and fatigue present unique barriers to effective oral care, making both preventative strategies and practical adaptations essential [1, 5, 23, 24, 28].

Routine oral care recommendations are broadly aligned across sources. These include brushing with soft‐bristled or electric toothbrushes, using low‐foaming toothpaste, and cleaning the tongue, gums and buccal mucosa to reduce microbial load [1, 23, 24, 28]. Regular dental reviews are advised regardless of oral feeding status, as tube‐fed individuals remain at risk for oral disease [29].

Guidance emphasises the use of assistive tools to adapt hygiene practices to the physical limitations caused by MND. Leaflets suggest oroswabs, finger brushes, bite blocks, and high‐speed suction devices to aid caregivers in providing safe and comfortable oral care [26, 28]. For patients with xerostomia, products such as artificial saliva and oral moisturisers are recommended to alleviate dryness and enhance oral comfort [27, 30].

Accessibility of dental services was a recurring theme. Recommendations include flexible appointment scheduling, wheelchair‐accessible clinics, and the use of specialised dental equipment to accommodate patients with limited mobility or fatigue [24, 28].

As with other aspects of care, a multidisciplinary and person‐centred approach is strongly endorsed. Integration of dental professionals, speech, and language therapists, and MND care teams is essential to ensure that oral hygiene plans are both effective and tailored to the individual's stage of disease and functional ability [1, 23, 26, 27].

#### 3.2.3 Dysphagia Management in Motor Neurone Disease (MND)

Dysphagia, resulting from progressive bulbar muscle weakness, is a common and serious concern in MND, contributing to malnutrition, dehydration, and aspiration pneumonia. Both clinical guidelines and patient information leaflets underscore the importance of early identification, individualised care planning and multidisciplinary collaboration in its management [1, 5, 22, 23, 24, 25, 28].

Dietary modification was universally recommended as a first‐line approach. This included transitioning to softer, pureed foods and thickened liquids to reduce aspiration risk and maintain adequate nutrition [1, 23, 24, 25, 28]. The NICE and EAN guidelines also advocate for timely consideration of gastrostomy, particularly when oral intake becomes unsafe or insufficient [1, 23]. These decisions are ideally made in consultation with the patient and family, respecting autonomy and ensuring care remains person‐centred.

Information leaflets expand on these clinical recommendations by offering practical strategies for safe eating. These include postural adjustments during meals, the use of adaptive feeding equipment, and stepwise guidance for educating carers on modified texture diets and aspiration precautions [5, 24, 28]. The psychosocial impacts of dysphagia, such as isolation and anxiety during mealtimes, are also acknowledged, with some resources recommending holistic care planning to address emotional well‐being [29, 30].

Multidisciplinary involvement, particularly from speech and language therapists, dietitians and nurses, was consistently emphasised to ensure regular reassessment and dynamic adjustment of feeding strategies as the disease progresses [5, 22, 23, 26]. While some guidelines focus more extensively on saliva control within the dysphagia context [22], others provide detailed frameworks for individualised nutritional management and decision‐making regarding artificial feeding [23].

## 4 Discussion

This scoping review mapped existing evidence on oral health care services and practices for individuals with Motor Neurone Disease (MND), identifying barriers and facilitators to maintaining oral hygiene and examining the extent to which oral health is integrated within multidisciplinary care. Eleven primary studies and eleven grey literature sources (three clinical guidelines and eight information leaflets) were included. The findings indicate that oral health was an overlooked but essential aspect of MND management. Poor oral health was linked to pain, malnutrition, aspiration pneumonia and diminished quality of life, whereas caregiver involvement, multidisciplinary collaboration, and adaptive oral care tools such as electric toothbrushes and suction devices supported better outcomes. Collectively, the results emphasize the need to view oral health as an integral component of holistic MND care.

A key strength of this review lies in its comprehensive scope. By including both peer‐reviewed and grey literature, it captured the breadth of existing knowledge and practice while reflecting real‐world experiences of patients and caregivers. The use of systematic search methods, duplicate screening, and independent data extraction enhanced the reliability and reproducibility of findings. However, the evidence base remains small and heterogeneous. Most included studies were case reports or small cross‐sectional surveys, limiting generalisability. Few evaluated interventions or reported long‐term outcomes, and the quality of grey literature was variable, with limited methodological transparency. As is typical of scoping reviews, no formal risk‐of‐bias assessment was undertaken, and unpublished or ongoing work may have been missed.

The findings align with broader research showing that oral health is often undervalued in neurodegenerative disease management. As observed in Parkinson's disease and dementia, oral care in MND is reactive, addressing complications only once pain, infection, or aspiration risk emerges [6, 15, 18]. The association between poor oral hygiene and aspiration pneumonia supports previous work demonstrating the systemic consequences of oral disease [7, 13, 17]. Progressive functional decline and bulbar weakness reduce patients' capacity for independent oral care, increasing reliance on carers who often lack formal training. This dependence contributes to inconsistent oral hygiene routines and emotional strain for both patients and caregivers. The limited participation of dental professionals in multidisciplinary teams reflects a systemic challenge across neurological conditions [8, 10].

Despite these shortcomings, several examples demonstrate promising practice. In Sweden and Australia, the integration of dental hygienists and domiciliary dental services into MND care pathways has been shown to improve accessibility and outcomes [15, 16]. Innovative clinical approaches such as antimicrobial photodynamic therapy [11] and customised oral appliances for managing drooling or self‐inflicted trauma [12, 20] illustrate clinical adaptability, though their effectiveness has yet to be tested in larger studies. Together, these findings reinforce that oral health is not peripheral but central to maintaining comfort, nutrition and respiratory safety for people living with MND.

In practice, oral health should be explicitly embedded within multidisciplinary MND frameworks [14, 19]. Routine oral assessments should accompany nutritional and respiratory reviews, with clear referral pathways to community dental services. Existing clinical guidelines [1, 22, 23] require expansion to include detailed oral‐care recommendations, defined dental roles within care teams, and structured caregiver training. Workforce development should prioritise education on adaptive oral‐care tools, safe saliva management, and early recognition of oral pathology. Extending national programmes such as Mouth Care Matters [9] to include progressive neurological conditions could improve consistency and equity of care. Commissioning domiciliary or mobile dental services would further reduce preventable oral morbidity among those unable to attend clinics.

In conclusion, this review highlights that although oral health significantly influences comfort, safety, and dignity for individuals with MND, it remains inadequately addressed in current models of care. The evidence points to an urgent need to integrate dental expertise into multidisciplinary teams and to establish national oral‐care standards tailored to MND. Future research should progress beyond descriptive studies towards evaluating interventions and long‐term outcomes. Embedding oral health within MND care frameworks will bridge the divide between dental and medical disciplines, promoting truly comprehensive care and improving quality of life for those affected by this progressive condition. The findings of the review will also support a larger study exploring the oral health experiences of people with MND and inform the development of an evidence‐based oral health care pathway and a self/carer‐care toolkit.

## Funding

This work was supported by Motor Neurone Disease Association (Glenny/Oct23/2334‐794).

## Conflicts of Interest

The authors declare no conflicts of interest.

## Data Availability

The data supporting this scoping review consist entirely of information extracted from previously published resources, all of which are cited within the manuscript. No new datasets were generated or analyzed during the current study. However, the data that support the findings of this study are available from the corresponding author upon reasonable request.
